# Synthesis, Characterization, Semiempirical and Biological Activities of Organotin(IV) Carboxylates with 4-Piperidinecarboxylic Acid

**DOI:** 10.1155/2014/959203

**Published:** 2014-10-13

**Authors:** Shabbir Hussain, Saqib Ali, Saira Shahzadi, Saroj K. Sharma, Kushal Qanungo, Muhammad Shahid

**Affiliations:** ^1^Department of Chemistry, GC University, Faisalabad 38000, Pakistan; ^2^Department of Chemistry, Quaid-i-Azam University, Islamabad 45320, Pakistan; ^3^Department of Applied Sciences and Humanities, Faculty of Engineering and Technology, Mody Institute of Technology and Science, Deemed University, Lakshmangarh, Sikar, Rajasthan 332311, India; ^4^Department of Chemistry and Biochemistry, University of Agriculture, Faisalabad 38000, Pakistan

## Abstract

Organotin (IV) carboxylates with the general formulae R_2_Sn(Cl)L [R = Me (**1**), *n*-Bu (**2**), Ph (**3**)] and R_3_SnL [R = Me (**4**), Ph (**5**)] have been synthesized by the reaction of 4-piperidinecarboxylic acid (HL) with KOH and R_2_SnCl_2_ (R = Me, *n*-Bu, Ph)/R_3_SnCl (R = Me, Ph) in methanol under stirring conditions. The metal ligand binding site, structure, and stability of complexes have been verified by FT-IR, (^1^H, ^13^C) NMR, EI-MS technique, and semiempirical study. The FT-IR data indicate the bidentate chelating mode of the carboxylate ligand which is also confirmed by semiempirical study. In solution state, five and four coordinated geometry around tin was confirmed by NMR spectroscopy. The EI-MS data agreed well with the molecular structure of the complexes. Thermodynamic parameters and molecular descriptors were calculated by using semiempirical PM3 method. HOMO-LUMO calculations show that chlorodiorganotin complexes are more susceptible to nucleophilic attack as compared to triorganotin complexes. Computed negative heat of formation indicates that complexes **1**–**4** are thermodynamically stable. The organotin(IV) carboxylates displayed powerful antimicrobial activities against various strains of bacteria and fungi and their minimal inhibitory concentration were also evaluated. The complexes exhibited comparatively higher hemolytic activity as compared to free ligand.

## 1. Introduction

Organotin complexes are subject of great interest due to their structural diversities and broad range of applications in various fields [1]. Coordination of carboxylates to organotin residues offers the possibility of studying the variations of the coordination modes which include monodentate, chelate, or the more subtle bridging, which may give rise to oligomeric or polymeric structures [1, 2]. Organotin(IV) carboxylates have been used in silicone curing [3], formation of polyurethane [4], antifouling paints [5], and PVC stabilization [6]. Organotin(IV) carboxylates possess significant properties as antibacterial and antifungal agents and also as antitumor and anticancer drugs [7]. The antifungal, antibacterial, and antitumor activities of organotin(IV) carboxylates are essentially related to the number and nature of the organic groups attached to the central Sn atom [8]. In general, triorganotin(IV) compounds display better biological activity than their diorganotin and monoorganotin analogs. This has been attributed to their ability to bind proteins [9]. However, the role of the ligand, for the transportation of organotin(IV) moiety to the target area, where the organotin(IV) species perform its biocidal activity, cannot be ignored [10]. The synthesis of organotin complexes in research area is of increased interest for inorganic, pharmaceutical, and medicinal chemistry as an approach to the development of new drugs [11, 12].

Research on the structure of organotin(IV) carboxylates continues and, at the same time, some new applications of high importance are being discovered which are relevant to ecological medicinal applications. The increasing interest in the chemistry of organotin(IV) compounds has led to the extended studies on their reactions with different biomolecules [13].

On the other hand, organotin(IV) compounds have been tested for their* in vitro *activity against a large variety of tumor cell lines [14] and have been found to be as effective as or better than traditional heavy metal anticancer drugs such as cisplatin.

Keeping in view the great importance of organotin chemistry and in continuation of our previous work [15–17], we report here the synthesis, characterization, semiempirical study, and biological activities of organotin carboxylates with 4-piperidinecarboxylic acid.

## 2. Experimental

### 2.1. Materials and Methods

Dimethyltin dichloride, dibutyltin dichloride, diphenyltin dichloride, trimethyltin chloride, and triphenyltin chloride were purchased from Sigma-Aldrich (USA) and used without any purification. 4-Piperidinecarboxylic acid was purchased from Merck (Germany). AR grade solvents of Merck (methanol), Lab-scan (DMSO), and Riedel-de Haen (petroleum ether) origin were used. The solvents were dried before use by standard procedures [18]. The samples were taken in capillary tubes and melting points were measured by an electrochemical melting point apparatus Stuart SMP3 and are uncorrected. Infrared spectra were recorded by a Perkin-Elmer-1000 FTIR spectrophotometer in the range of 4000–250 cm^−1^ as KBr/CsBr pellets. The ^1^H and ^13^C NMR spectra were recorded by Bruker ARC 300 MHz-FT-NMR spectrometer. The percentage composition of C, H, and N was determined by using CHNS-932 Leco (USA). The complexes were modeled by MOPAC 2007 [19] program in gas phase using PM3 method [20, 21]. Selected parts of the complexes not containing the metal ion were preoptimised using molecular mechanics methods. Several cycles of energy minimization were carried for each of the complexes. Geometry was optimized using Eigen Vector following. The root mean square gradient for complexes was less than one. Self-consistent field was achieved in each case.

Antimicrobial activities of the ligand and complexes were tested against bacteria (*Escherichia coli, Bacillus subtilis, Staphylococcus aureus*, and* Pasteurella multocida*) and fungi (*Alternaria alternata, Ganoderma lucidum, Penicillium notatum, Trichoderma harzianum*, and* Aspergillus niger*) by disc diffusion method [22] and minimum inhibitory concentration (MIC) [23]. The activities were performed in an incubator (Sanyo, Germany) and sterilized in an autoclave (Omron, Japan). The minimum inhibitory concentration was determined in a Micro Quant apparatus (BioTek, USA). Streptomycin and fluconazole were used as standard drugs for antibacterial and antifungal screening tests, respectively. The* in vitro* hemolytic bioassay [24] of the complexes was reported with respect to the triton X-100 as positive control and PBS as negative control.

### 2.2. General Procedure for the Synthesis of Complexes** 1**–**5**

4-Piperidinecarboxylic acid (1 mmol) and KOH (1 mmol) were stirred together in methanol (50 mL) for 1 hr in a 100 mL round bottom flask at room temperature. Then, R_2_SnCl_2_/R_3_SnCl (1 mmol) was added as solid in portions and reaction mixture was continuously stirred for 5 hr (Equations (1) and (2) in Figure 5). The precipitated KCl was filtered off and solvent was evaporated through rotary evaporator under reduced pressure. The product obtained was recrystallized from methanol and petroleum ether (2 : 1).

## 3. Results and Discussion

The complexes are solid having sharp melting points and are soluble in common organic solvents. The physical data is given in Table 1.

### 3.1. IR Spectroscopy

Infrared spectra of the organotin(IV) complexes provide valuable information regarding the structure of compounds and coordination geometry of the metal in the solid state. Infrared spectra were recorded as KBr/CsBr disc in the range of 4000–250 cm^−1^ and the important bands are given in Table 2. The peculiar feature of the IR spectra of the complexes is the absence of *ν*OH stretching vibration of the free carboxylic acid at 3639 cm^−1^ due to deprotonation for coordination with tin(IV).

IR spectroscopy supported the noninvolvement of amino nitrogen of the ligand** HL** with tin because vibrational frequency of *ν*NH was not shifted to a considerable extent in complexes** 1**–**5** as compared to the free ligand** HL**; these findings were further verified by ^1^H and ^13^C NMR spectroscopy. The mode of tin carboxylate interaction was predicted from Δ*ν* = *ν*COO_(asym)_ − *ν*COO_(sym)_ value; Δ*ν* in the complexes lie in the range of 154–184 cm^−1^ suggesting bidentate binding mode of the carboxylate group [25, 26]. Bidentate mode owes to the donation of electron density from C=O group to the tin. Bands for Sn-C vibration of organotin moieties were observed in the range 519–554 cm^−1^ [complexes** 1**–**3**] and in the range 264–279 cm^−1^ [complexes** 4** and** 5**]. The appearance of new bands in the range 442–451 cm^−1^ and 318–375 cm^−1^ was assigned to Sn–O and Sn–Cl bond formation which further confirms complexation.

### 3.2. ^1^H NMR Spectroscopy

The ^1^H NMR data is given in Table 3. The signals were assigned by their distinct multiplicity patterns, resonance intensities, coupling constants, and tin satellites. The number of protons found by integration of peaks in the spectra agreed well with those calculated from the expected composition.

The absence of signal for carboxylic (–COOH) proton at 11.6 ppm of the free ligand in the spectra of complexes verified the coordination through deprotonated carboxylate anions. The amino proton of the free ligand** HL** does not show significant shift in the complexes** 1**–**5**, demonstrating the noninvolvement in coordination with tin and supporting the IR findings. The chemical shifts were assigned to the protons of alkyl- or aryltin moieties in complexes according to literature [27].

Table 4 represents the coupling constants (^*n*^ *J*) obtained from resolved satellites and the calculated C−Sn−C bond angles (*θ*) in solution state of di- and trimethyltin(IV) derivatives. The data strongly supports five and four coordinated geometry around tin [28]. Despite the complex pattern of di-*n*-butyl fragments in the spectrum of complex** 2**, a clear triplet due to terminal methyl group appeared at 0.89 ppm with ^3^ *J*(^1^H, ^1^H) = 7.2 Hz [15]. Ortho protons absorbed downfield as compared to meta and para protons in phenyltin(IV) derivatives [16]. ^1^H NMR data for the phenyltin(IV) derivatives** 3** and** 5** described the existence of ortho (*β*) protons in the range 7.81–7.89 ppm as multiplet, while a multiplet in the range of 7.35–7.52 ppm and 7.50–7.62 ppm was assigned to meta (*γ*) and para (*δ*) protons, respectively. The ^2^ *J*[^119^Sn, ^1^H] values for complexes** 1** and** 4 **are 90 and 52 Hz, respectively [16]. As far as the piperidine ring of the ligand is concerned, resonances for equatorial protons was assigned downfield as compared to the corresponding axial ones, according to literature [17].

### 3.3. ^13^C NMR Spectroscopy

The ^13^C NMR spectra of the complexes** 1**–**5** were recorded in deuterated DMSO and the resonances were assigned by comparison with the results obtained from incremental method [29]. The ^13^C NMR data is given in Table 5. The carboxylate carbon exhibited downfield chemical shift in the range 179.1–179.8 ppm in complexes** 1**–**5**. The resonance in the range 42.1–42.8 ppm was assigned to carbon atom (labeled as 2) present in the nearest neighborhood of carboxylate group, while signals at 27.2–27.9 ppm and 51.2–51.8 ppm were allocated for the carbon atoms labeled as 3,3′ and 4,4′, respectively. Dimethyltin(IV) moiety demonstrated chemical shift at 24.6 ppm in complex** 1** while complex** 2 **exhibited signals at 25.7, 27.7, 26.2, and 14.2 for *α*, *β*, *γ*, and *δ*, carbon atoms, respectively, for butyl fragments.

### 3.4. Mass Spectrometry

The electron ionization mass spectra (EI-MS) were recorded for complexes** 2**,** 3,** and** 5**. The data is given in Table 6. Molecular ion peak was only observed in complex** 5** and was not displayed in the mass spectra of organotin(IV) derivatives** 2** and** 3 **[30]. Complex** 2** is primarily dissociated into chlorodibutytin (*m*/*z* = 269 (3%)) fragment and the deprotonated carboxylate ligand (*m*/*z* = 128 (3%)) or the primary fragmentation may eliminate two butyl radicals and one HCl molecule to produce [C_6_H_9_NO_2_Sn]^+^ ion (*m*/*z* = 247 (9%)); the fragmentation is terminated with the appearance of butyl ions (*m*/*z* = 57 (100%)) as a base peak. The primary fragmentation in complex** 3** occurs by the loss of a phenoxide ion showing a signal for M-93 ion or it may split the parent molecule into the deprotonated carboxylate ligand at *m*/*z* = 128 (14%) and the chlorodiphenyltin(IV) moiety at *m*/*z* = 309 (1%) thus strongly supporting the chlorodiorganotin(IV) product. The fragmentation is terminated with the decomposition of phenyl group to prouce C_4_H_3_ cation at* m/z* 51 (56%). In triphenyltin(IV) derivative** 5**, two peaks with *m*/*z* = 479 (26%) and *m*/*z* = 480 (30%) correspond to the molecular ion (M^+^) and M+1 ions, respectively. There are two modes of fragmentation; the dissociation may proceed* via* the loss of phenyl radicals step by step to yield the deprotonated carboxylate ligand, [C_12_H_10_Sn]^+^ (*m*/*z* = 128 (100%)) which then lose an oxygen atom to form [C_6_H_10_NO]^+^ ion (*m*/*z* = 112 (23%)) as end product or it may go ahead by the loss of a ligand fragment [C_6_H_10_NO^•^] to yield triphenyltin cation [C_18_H_15_Sn]^+^ (*m*/*z* = 351 (77%)) which is finally degraded into [Sn]^+^ ion (*m*/*z* = 120 (1%)) after successive removal of the phenyl radical in three steps.

### 3.5. Semiempirical Study

In complexes** 1**–**4**, the ligand binds in bidentate fashion to the Sn(IV) atom. The three-organic groups/two organic groups, one Cl group, and the ligand are arranged in distorted trigonal bipyramidal geometry. One of the Sn–O bond lengths is shorter than the other and is consistent with literature [15]. The Sn–C and Sn–Cl bond lengths are close to reported values [15]. The O–Sn–O and C–Sn–C bond angles are also consistent with earlier reported structures [15]. All other bond lengths and angles (Table 7) are also comparable to literature values [15].

**Figure 1:**
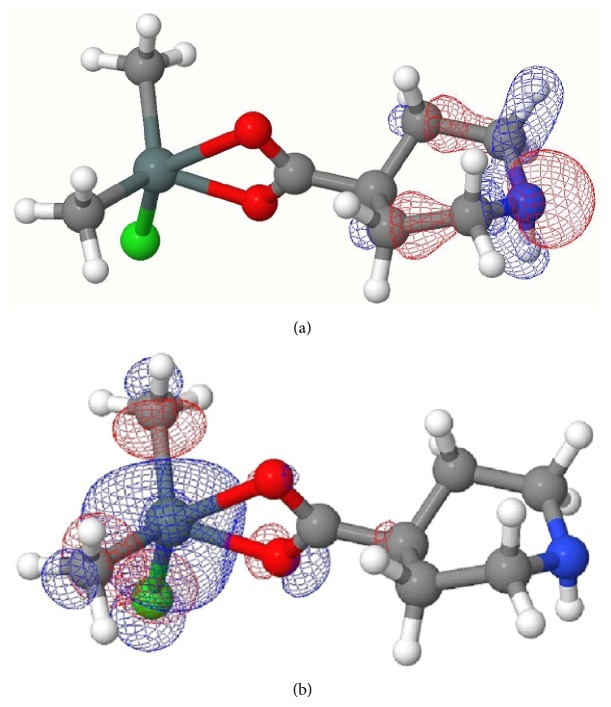
(a) HOMO of complex** 1**; (b) LUMO of complex** 1**.

**Figure 2:**
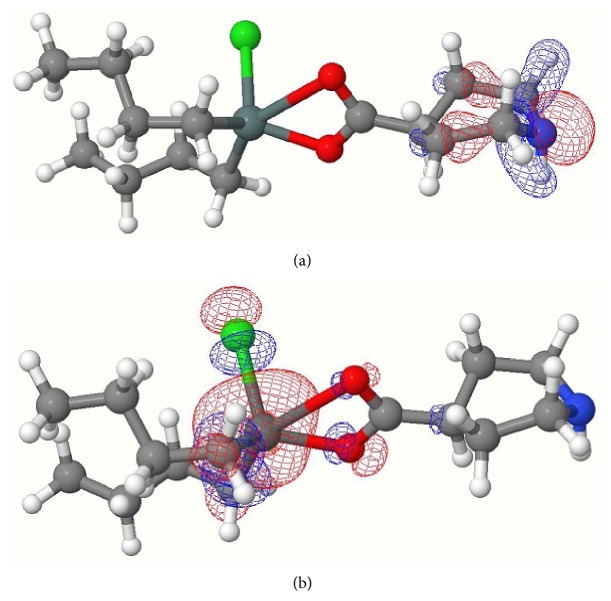
(a) HOMO of complex** 2**; (b) LUMO of complex** 2**.

**Figure 3:**
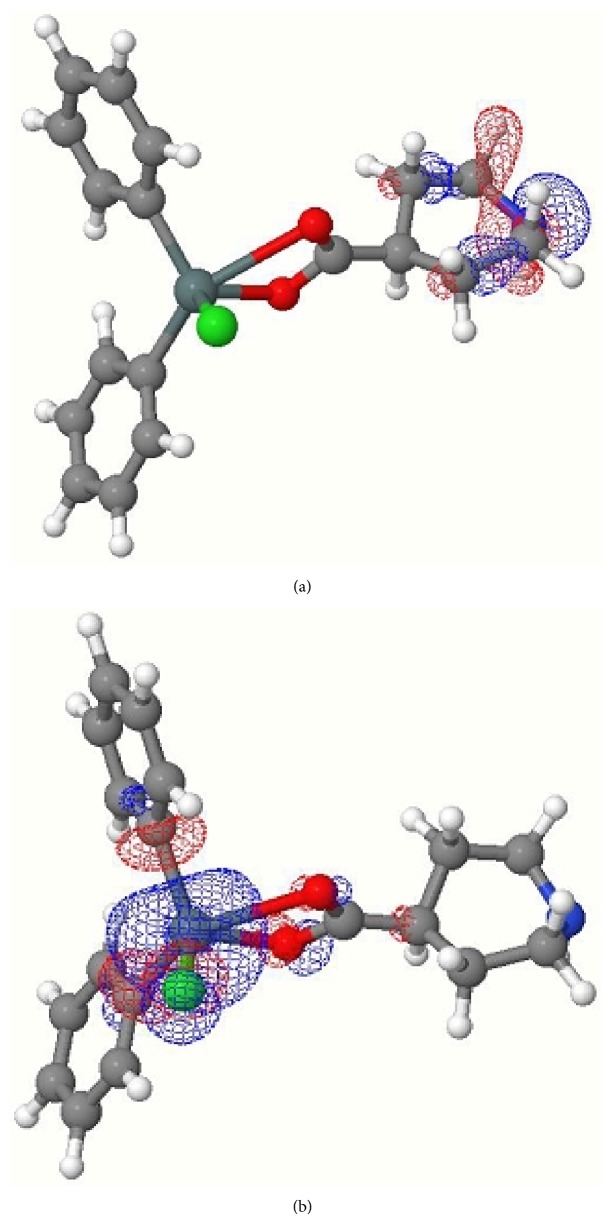
(a) HOMO of complex** 3**; (b) LUMO of complex** 3**.

**Figure 4:**
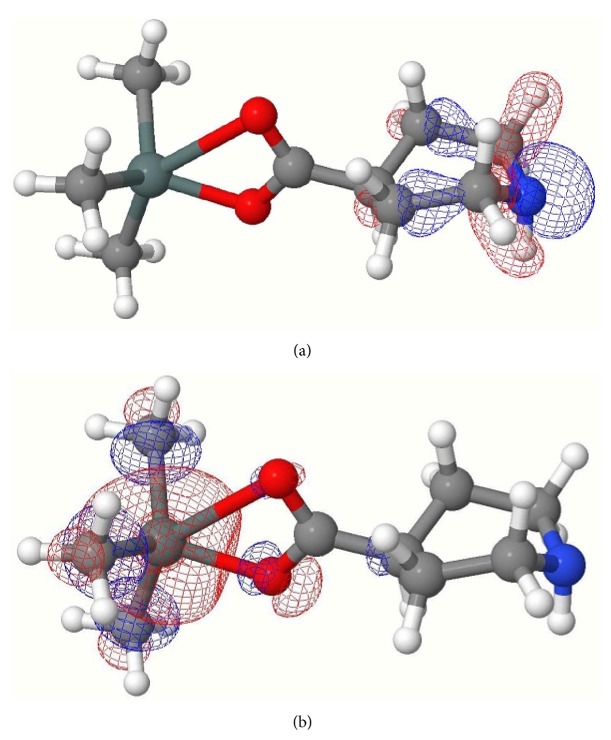
(a) HOMO of complex** 4**; (b) LUMO of complex** 4**.

**Figure 5:**
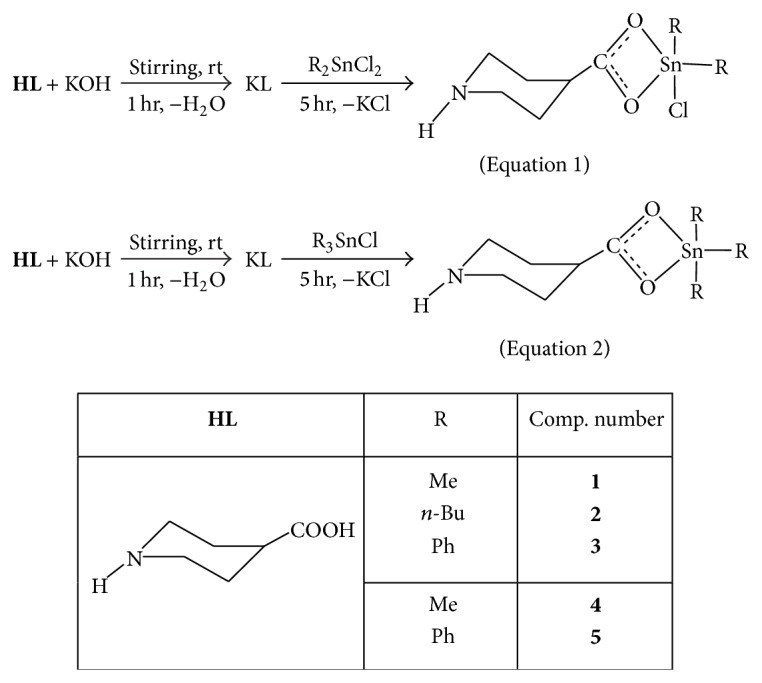


Computed negative heats of formation indicate that all complexes are thermodynamically stable (Table 8). The calculated HOMO and LUMO orbitals of complexes** 1**–**4** are shown in Figures 1, 2, 3, and 4. Notice in all the complexes without exception that the HOMO orbitals are localized on the piperidine ring, while the LUMO orbitals are localized on tin, carboxylates oxygens, and carbon atoms bonded to tin. The calculated HOMO and LUMO energies are given in Table 9. Though semiempirical method overestimates the HOMO and LUMO energies, the trends in variations are noteworthy. Substitution of an alkyl/aryl group in the organotin moieties by a chloride group lowers both the HOMO and LUMO energies in all the mono chlorosubstituted complexes. This lowering is more for LUMO than for HOMO, the overall effect being that the HOMO-LUMO gap is lower in the mono chlorosubstituted complexes. *E*_HOMO_ is associated with the ability of the molecule to donate electrons, (−Ionization Potential), and in this respect all the complexes have comparable electron donating abilities, (the effect of Me/Cl/Bu/Ph substituent is small). A comparison of *E*_HOMO_ values shows that, in each case, the alkyl/aryl organotin complexes are predicted to have a slightly higher electron donating ability than the mono chlorosubstituted one. *E*_LUMO_ on the other hand reflects the ability of a molecule to accept electrons (−Electron Affinity), and LUMO energy of the mono chlorosubstituted organotin moieties is nearly three-four times lower than that of the unsubstituted ones. This indicates that mono chlorosubstituted complexes have a better ability to accept electrons and are reflected by their higher electrophilicity values (*ω* = *μ* ^2^/2*η*) [31] in each case (Table 9). A comparison of global hardness (*η* = *I* − *A*/2) values [32] (Table 9) shows that alkyl/aryl substituted complexes are harder than the mono chlorosubstituted tin compounds. A similar conclusion can be drawn from a comparison of global softness values (*S* = 1/2*η*) [33] (Table 9). A comparison of the chemical potential values *μ* = −(*I* + *A*)/2 (Table 9) [34] shows that the presence of electron donating alkyl/aryl groups increases the chemical potential and* vice versa*.

### 3.6. Antimicrobial Activity

The ligand** HL** and complexes** 1**–**5** were screened to check their* in vitro* response against various strains of bacteria (*Escherichia coli, Bacillus subtilis, Staphylococcus aureus*, and* Pasteurella multocida*) and fungi (*Alternaria alternata, Ganoderma lucidum, Penicillium notatum, Trichoderma harzianum*, and* Aspergillus niger*). The data is given in Tables 10 and 11. It was found that the organotin(IV) complexes possessed significantly higher activity towards the tested organisms than the free ligands as reported earlier [12, 14, 35]. The coordination of the ligand with chlorodi- or triorganotin(IV) moieties has appreciably enhanced the activities of the product complexes. A close relationship was observed between structure and activity of the synthesized complexes; biological (antibacterial/antifungal) activities of the organotin(IV) complexes** 1**–**5** were varied according to their substitution pattern at tin. The inhibitory action of organotin(IV) compounds is mainly due to their ability to interact with DNA and protein. The MIC data (Tables 12 and 13) also supports that the complexes displayed strong growth inhibitory effect against all microbes in contrast to the biologically inactive ligand [36].

### 3.7. Hemolytic Activity

Hemolytic activity was studied because, even if a synthesized compound possesses potent antimicrobial activities, its use in medicine will be impossible in the presence of hemolytic effects. Thus,* in vitro* hemolytic bioassays of the synthesized complexes was carried out with human red blood cells and the average lysis was observed with respect to the triton X-100 as positive control (100% lysis) and PBS as negative control (0% lysis). The results are given in Table 14.

The lowest activity (10.58%) was observed for complex** 3**; however, this activity was found comparatively higher than free ligand** HL**. The highest value (26.85%) was recorded for the dimethyltin(IV) complex** 1**. However, it is worth mentioning that all the synthesized complexes possessed hemolytic activity which is very much lower as compared to triton X-100 and higher than PBS.

## 4. Conclusion

FTIR data indicates the bidentate chelating mode of carboxylate group which is confirmed by semiempirical study. NMR data shows 5- and 4-coordinated geometry in solution state. The EI-MS fragmentation patterns agreed well with the molecular structures of the complexes. HOMO-LUMO calculations show that chlorodiorganotin complexes are more susceptible to nucleophilic attack as compared to triorganotin complexes. Computed negative heat of formation indicates that complexes** 1**–**4** are thermodynamically stable and chemically inert. The complexes exhibited significant antimicrobial activities. The hemolytic activity of complexes shows that the complexes exhibit comparatively higher hemolytic activity as compared to the free ligand.

## Figures and Tables

**Scheme 1:**
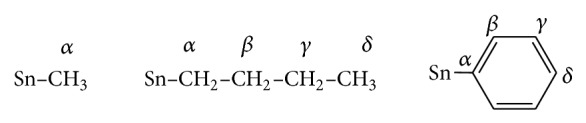


**Table 1:** Physical data of organotin complexes **1–5**.

Comp. number	Molecular formula	Mol. Wt	Yield (%)	m.p (°C)	Elemental analysis Calcd. (found)		
					%C	%H	%N
**HL**	C_6_H_11_NO_2_	129.16	—	300	—	—	—
**1**	C_8_H_16_NO_2_SnCl	312.38	84	220	30.73 (30.77)	5.12 (5.16)	4.48 (4.44)
**2**	C_14_H_28_NO_2_SnCl	396.54	84	233	42.36 (42.41)	7.06 (7.02)	3.53 (3.57)
**3**	C_18_H_20_NO_2_SnCl	436.52	82	272	49.48 (49.52)	4.58 (4.62)	3.20 (3.24)
**4**	C_9_H_19_NO_2_Sn	291.96	85	261	36.99 (36.95)	6.50 (6.54)	4.79 (4.83)
**5**	C_24_H_25_NO_2_Sn	478.17	81	245	60.22 (60.26)	5.22 (5.26)	2.92 (2.96)

**Table 2:** IR data^a^ (cm^−1^) of organotin(IV) complexes **1–5**.

Comp. number	*ν*OH	*ν*NH	*ν*COO		Δ*ν*	*ν*Sn–C	*ν*Sn–O	*ν*Sn–Cl
			asym	sym				
**HL**	3639 b	3456b	1671w	1398s	273	—	—	—
**1**	—	3459m	1622s	1438s	184	543b	450m	375s
**2**	—	3457b	1629s	1483m	146	519w	449w	328s
**3**	—	3461b	1606s	1452w	154	279s	442b	318b
**4**	—	3462b	1601s	1441w	161	554s	451m	—
**5**	—	3456b	1551s	1370m	181	264s	447m	—

^a^s: strong; m: medium; w: weak; b: broad.

**Table 3:** ^
1^H NMR data^a^ of organotin complexes **1–5**.

	Proton number	Chemical shift (ppm)					
		**HL**	**1**	**2**	**3**	**4**	**5**
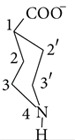	1	2.32–2.41m	2.74–2.81m	2.90–2.96m	2.61–2.65m	2.36–2.43m	2.73–2.81m
	2, 2′(a)	1.83–1.89m	1.82–1.98m	1.86–1.90m	1.85–1.93m	1.78–1.88m	1.80–1.97m
	2, 2′(e)	2.12–2.17m	2.11–2.19m	2.11–2.18m	2.12–2.17m	2.05–2.11m	2.08–2.21m
	3, 3′(a)	2.74–2.79m	3.05–3.13m	2.88–2.92m	3.07–3.12m	2.97–3.05m	3.01–3.13m
	3, 3′(e)	3.23–3.35m	3.36–3.41m	3.15–3.22m	3.35–3.38m	3.32–3.35m	3.35–3.43m
	4	2.50s	2.58s	2.52s	2.57s	2.59s	2.58s

Sn–R	*α*		0.78s [90]	1.19–1.37m	—	0.4s [52]	—
	*β*		—	1.59–1.61m	7.85–7.89m	—	7.81–7.88m
	*γ*		—	1.40–1.56m	7.35–7.49m	—	7.42–7.52m
	*δ*		—	0.89t (7.2)	7.51–7.59m	—	7.50–7.62m

^a^ ^*n*^ *J*[^119^Sn, ^1^H] and ^*n*^ *J*(^1^H, ^1^H) are listed in square brackets and parenthesis, respectively; multiplicity is given as s: singlet, t: triplet, and m: multiplet.

See Scheme 1.

**Table 4:** (C–Sn–C) angles (°) based on NMR parameters.

Comp. number	^ 2^ *J*(^119^Sn, ^1^H) (Hz)	Angle, *θ*
**1**	90.0	135.5
**4**	52	109.2

**Table 5:** ^
13^C NMR data^a^ of organotin complexes **1–5**.

	Carbon number	Chemical shift (ppm)					
		**HL**	**1**	**2**	**3**	**4**	**5**
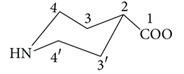	1	178.2	179.5	179.1	179.6	179.8	179.7
	2	42.6	42.1	42.8	42.4	42.6	42.8
	3, 3′	24.2	27.3	27.7	27.2	27.9	27.2
	4, 4′	51.1	51.3	51.6	51.5	51.8	51.2

Sn–R	*α*	—	24.6 [593.1, 567.3]	25.7 [541.1, 516.3]	135.2 [56.4]	−2.0 [395.6]	138.06 [583.1, 557.3]
	*β*	—	—	27.7 [39.0]	141.2		128.99 [61.4, 59.0]
	*γ*	—	—	26.2 [117.3, 112.6]	129.1 [88.2]		136.57 [44.3]
	*δ*	—	—	14.2	130.2 [56.6]		129.94 [12.5]

^a^Chemical shifts (*δ*) in ppm. ^*n*^ *J*[^119/117^Sn, ^13^C] in Hz is listed in square brackets.

See Scheme 1.

**Table 6:** Mass spectral data of organotin complexes **2**, **3,** and **5**.

Comp. number	*m*/*z* (%)
**2**	[C_14_H_28_NO_2_SnCl]^+^ 397 (n.o)^#^, [C_8_H_18_ClSn]^+^ 269 (3), [C_6_H_9_NO_2_Sn]^+^ 247 (9), [C_4_H_9_ClSn]^+^ 212 (8), [C_4_H_9_Sn]^+^ 177 (2), [HO_2_Sn]^+^ 153 (12), [C_6_H_10_NO_2_]^+^ 128 (3), [Sn]^+^ 120 (2), [C_5_H_8_N]^+^ 82 (3), [C_4_H_9_]^+^ 57 (100).

**3**	[C_18_H_20_NO_2_SnCl]^+^ 437 (n.o)^#^, [C_12_H_15_ClNOSn]^+^ 344 (1), [C_12_H_10_ClSn]^+^ 309 (1), [C_6_H_10_ClNOSn]^+^ 267 (3), [C_6_H_8_ClNOSn]^+^ 265 (2), [C_6_H_5_ClSn]^+^ 232 (3), [C_6_H_5_Sn]^+^ 197 (3), [C_12_H_10_]^+^ 154 (9), [C_6_H_10_NO_2_]^+^ 128 (14), [C_6_H_10_NO]^+^ 112 (3), [C_6_H_6_]^+^ 78 (100), [C_6_H_5_]^+^ 77 (35), [C_3_HN]^+^ 51 (56).

**5**	[C_24_H_25_NO_2_Sn]^+^ 479 (26)^#^, [C_24_H_25_NO_2_Sn]^+^ 480 (30), [C_18_H_20_NO_2_Sn]^+^ 402 (3), [C_18_H_15_Sn]^+^ 351 (77), [C_12_H_15_NO_2_Sn]^+^ 325 (1), [C_12_H_10_Sn]^+^ 274 (1), [C_6_H_5_Sn]^+^ 197 (1), [C_12_H_10_Sn]^+^ 128 (100), [Sn]^+^ 120 (1), [C_6_H_10_NO]^+^ 112 (23).

^#^Molecular ion peak (M^+^); n.o: not observed.

**Table 7:** Computed structural parameters of complexes **1–4**.

Comp. number	**1**	**2**	**3**	**4**
Sn–O bond lengths (Å)	2.02, 2.61	2.02, 2.66	2.00, 2.65	2.03, 2.73
Sn–C bond lengths (Å)	2.08, 2.08	2.12, 2.13	2.05, 2.05	2.10, 2.10, 2.11
Sn–Cl bond length (Å)	2.36	2.37	2.35	—
O–Sn–O (°)	51.8	50.9	51.1	49.3
C–Sn–C (°)	119.3	115.6	114.6	109.9, 109.9, 114.2

**Table 8:** Computed thermodynamic parameters (at 298 K) of complexes **1–4**.

Comp. number	**1**	**2**	**3**	**4**
Heat of formation (KCal/mole)	−137.453	−161.812	−65.424	−109.346
Enthalpy (KCal/mole-K)	9.308	11.340	11.000	9.608
Entropy (KCal/mole-K)	0.117	0.130	0.128	0.118
Heat capacity (Cp) (Cal/mole-K)	54.555	72.716	71.764	57.727

**Table 9:** Computed molecular descriptors of complexes **1–4**.

Comp. number	**1**	**2**	**3**	**4**
HOMO energy (eV)	−9.582	−9.527	−9.510	−9.408
LUMO energy (eV)	−1.483	−1.524	−1.556	−0.387
HOMO-LUMO (eV)	8.099	8.003	7.954	9.021
Dipole moment (debye)	3.282	4.509	4.477	1.668
Global hardness (*η*, eV)	4.049	4.001	3.977	4.510
Global softness (*S*, eV^−1^)	0.123	0.124	0.125	0.110
Chemical potential (*μ*, eV)	−5.532	−5.525	−5.533	−4.897
Electophilicity (*ω*)	3.77	3.81	3.84	2.65

**Table 10:** Antibacterial activity data^a, b^ of organotin complexes **1–5**.

Comp. number	Bacterial inhibition zone (mm)			
	*E. coli *	*B. subtilis *	*S. aureus *	*P. multocida *
**HL**	—	—	—	—
**1**	30^a^ ± 0.14	20^bc^ ± 0.07	30^ab^ ± 0.28	22^b^ ± 0.20
**2**	26^ab^ ± 0.22	25^ab^ ± 0.19	30^ab^ ± 0.21	28^ab^ ± 0.31
**3**	28^ab^ ± 0.30	20^bc^ ± 0.14	30^ab^ ± 0.29	27^ab^ ± 0.14
**4**	14^c^ ± 0.12	14^c^ ± 0.15	16^c^ ± 0.20	15^c^ ± 0.11
**5**	21^bc^ ± 0.13	19^bc^ ± 0.07	23^bc^ ± 0.12	25^ab^ ± 0.22
Streptomycin	30^a^ ± 0.17	31^a^ ± 0.28	31^ab^ ± 0.31	29^a^ ± 0.28

^a^Data are expressed as the mean ± standard deviation of samples analyzed individually in triplicate at *P* < 0.1.

^b^It refers that how does bacterial inhibition zones are being calculated.

**Table 11:** Antifungal activity data^a, b^ of organotin complexes **1–5**.

Comp. number	Fungal inhibition zone (mm)				
	*A. alternata *	*G. lucidum *	*P. notatum *	*T. harzianum *	*A. niger *
**HL**	—	—	—	—	—
**1**	14^c^ ± 0.15	21^c^ ± 0.20	14^c^ ± 0.11	13^c^ ± 0.07	25^c^ ± 0.23
**2**	20^bc^ ± 0.19	22^bc^ ± 0.13	19^bc^ ± 0.08	18^c^ ± 0.15	26^bc^ ± 0.23
**3**	22^bc^ ± 0.20	24^bc^ ± 0.14	28^bc^ ± 0.14	21^ab^ ± 0.18	26^bc^ ± 0.21
**4**	18^bc^ ± 0.11	23^bc^ ± 0.19	17^bc^ ± 0.07	17^bc^ ± 0.12	26^bc^ ± 0.15
**5**	25^bc^ ± 0.23	25^bc^ ± 0.21	28^bc^ ± 0.12	23^a^ ± 0.20	28^bc^ ± 0.23
Fluconazole	38^a^ ± 0.29	41^a^ ± 0.21	45^a^ ± 0.31	—	37^a^ ± 0.23

^a^Data are expressed as the mean ± standard deviation of samples analyzed individually in triplicate at *P* < 0.1.

^b^It refers that how does fungal inhibition zones are being calculated.

**Table 12:** MIC (bacterial) of organotin complexes **1–5**.

Comp. number	Minimum inhibitory concentration (*μ*g/well)			
	*E. coli *	*B. subtilis *	*S. aureus *	*P. multocida *
**HL**	—	—	—	—
**1**	50	25	25	—
**2**	12.5	1.56	7.81 × 10^−1^	7.81 × 10^−1^
**3**	12.5	25	12.5	50
**4**	50	—	50	—
**5**	12.5	12.5	12.5	12.5
Streptomycin	9.7 × 10^−2^	1.95 × 10^−1^	6.25	3.12

**Table 13:** MIC (fungal) of organotin complexes **1–5**.

Comp. number	Minimum inhibitory concentration (*μ*g/well)				
	*A. alternata *	*G. lucidum *	*P. notatum *	*T. harzianum *	*A. niger *
**HL**	—	—	—	—	—
**1**	—	6.25	25	7.81 × 10^−1^	3.12
**2**	1.56	7.81 × 10^−1^	7.81 × 10^−1^	3.90 × 10^−1^	7.81 × 10^−1^
**3**	7.81 × 10^−1^	1.95 × 10^−1^	1.95 × 10^−1^	9.76 × 10^−2^	3.12
**4**	12.5	3.12	12.5	7.81 × 10^−1^	3.12
**5**	9.76 × 10^−2^	3.12	2.44 × 10^−2^	2.44 × 10^−2^	3.90 × 10^−1^
Fluconazole	1.56	1.56	2.44 × 10^−2^	25	—

**Table 14:** Hemolytic activity of organotin complexes **1–5**.

Comp. number	% of hemolysis
**HL**	8.70 ± 0.03
**1**	26.85 ± 0.05
**2**	14.42 ± 0.05
**3**	10.58 ± 0.08
**4**	24.02 ± 0.03
**5**	20.93 ± 0.06
Triton X-100	99.53 ± 0.00
